# *Castanopsis lamontii* Water Extract Shows Potential in Suppressing Pathogens, Lipopolysaccharide-Induced Inflammation and Oxidative Stress-Induced Cell Injury

**DOI:** 10.3390/molecules24020273

**Published:** 2019-01-12

**Authors:** Ying Gao, Xinzhong Zhang, Junfeng Yin, Qizhen Du, Youying Tu, John Shi, Yongquan Xu

**Affiliations:** 1 Tea Research Institute Chinese Academy of Agricultural Sciences, Ministry of Agriculture, Hangzhou 310008, China; yinggao@tricaas.com (Y.G.); zxz.1982@163.com (X.Z.); 2 College of Agricultural and Food Sciences, Zhejiang A & F University, Linan 311300, China; qizhendu@163.com; 3 Department of Tea Science, Zhejiang University, Hangzhou 310058, China; 4 Guelph Food Research and Development Center, Agriculture and Agri-Food Canada, Guelph, ON N1G 5C9, Canada; johnshi2006@yahoo.ca

**Keywords:** *Castanopsis lamontii*, epicatechin, procyanidin B2, anti-bacteria, anti-inflammation, antioxidant

## Abstract

*Castanopsis lamontii* is traditionally used to prevent inflammatory diseases such as periodontitis and pharyngitis by residents in southwest China. However, little scientific evidence has been found to support this. In this research, the antibacterial activities of *Castanopsis lamontii* water extract (CLE) were assessed using the micro-dilution method. The anti-inflammatory and antioxidant activities of CLE were investigated in RAW264.7 cells. Key bioactive compounds in CLE were also explored. Results showed that CLE was capable of inhibiting the periodontitis pathogen *Porphyromonas gingivalis* and the pharyngitis pathogen β-hemolytic *Streptococcus*. It suppressed lipopolysaccharide-induced inflammation in RAW 264.7 cells via inactivating the TLR4/NF-κB pathway. Besides, it reduced oxidative stress-induced cell injury via scavenging reactive oxygen species. Chemical composition analysis revealed that CLE was rich in epicatechin and procyanidin B2. Further studies confirmed that epicatechin predominantly contributed to the antibacterial activities of CLE, while procyanidin B2 was mainly responsible for the anti-inflammatory activities of CLE. Both compounds contributed to the antioxidant activities of CLE. Acute oral toxicity tests proved that CLE was practically non-toxic. These results provide experimental evidences of the health-beneficial effects of CLE and may help promote the application of CLE in the food and health industries.

## 1. Introduction

**Figure 1:**
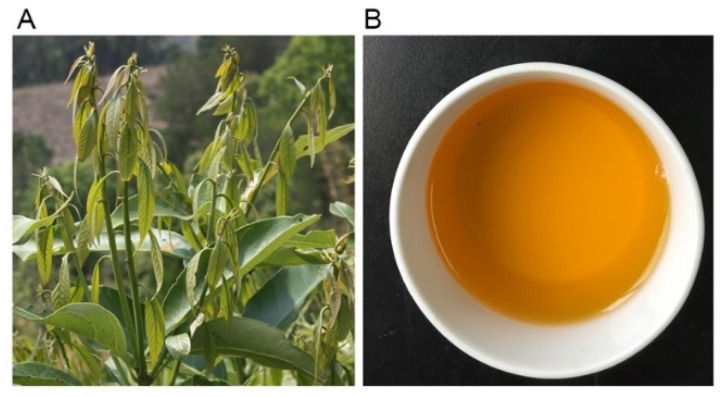
The buds of *Castanopsis lamontii* (**A**) and its infusion (**B**).

*Castanopsis lamontii* belongs to the *Fagaceae* family. The buds of *Castanopsis lamontii* (Figure 1) are traditionally applied as a herb to prevent inflammatory diseases such as periodontitis and pharyngitis. Residents in southwest China used to brew the buds and then drink the infusion to freshen the breath, protect teeth, and keep the pharynx healthy. Previous studies have shown that the leaves, fruits, and seeds of the *Fagaceae* family are rich in flavonoids, triterpene, triterpenoids, and polyphenols. Extracts from the *Fagaceae* family have been demonstrated to exhibit various biological activities. For example, the leaf extract from *Lithocarpus polystachyus* Rehd., containing trilobatin and phloridzin, promoted glycogen synthesis in T2DM mice [1]. The seed extract from *Lithocarpus dealbata* (Miq.) Rehder which was abundant in tannins, showed antidiarrheal potential in animal models [2]. Methanol extracts from the leaves, stem, and root barks of *Lithocarpus celebicus* showed broad-spectrum antibacterial activities [3]. However, the chemical composition of the buds of *Castanopsis lamontii* remains unclear and the physiological activities have not yet been demonstrated in the lab.

In this study, general chemical analysis was carried out to unveil the chemical composition of *Castanopsis lamontii* water extract (CLE). Ultra-performance liquid chromatography-quadrupole-time-of-flight/mass spectrometry (UPLC-Q-TOF/MS) analysis was used to identify the predominant compounds. The antibacterial, anti-inflammatory and antioxidant activities of CLE were determined to assess its ability to prevent periodontitis and pharyngitis. Key compounds contributed to the physiological activities of CLE were investigated. Acute oral toxicity tests were performed to evaluate its toxicity. The results will deepen the understanding of CLE and provide a theoretical basis for the application of this traditional herb in the food and health industries.

## 2. Results and Discussion

### 2.1. Main Chemical Composition of CLE

**Figure 2:**
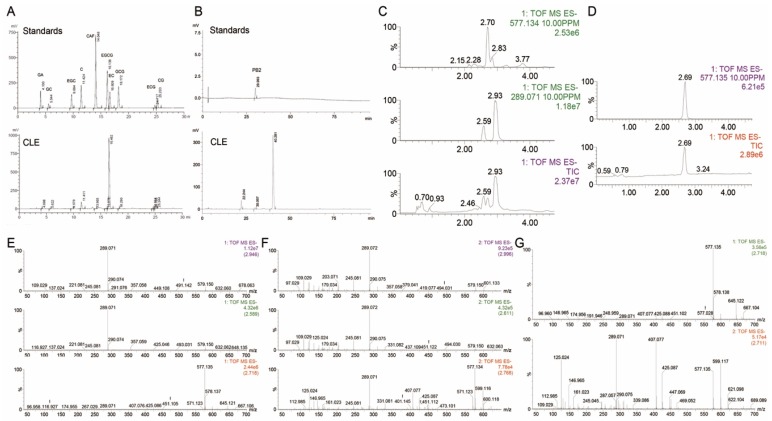
HPLC-UV and ultra-performance liquid chromatography-quadrupole-time-of-flight/mass spectrometry (UPLC-Q-TOF/MS) analysis of the *Castanopsis lamontii* water extract (CLE). (**A**) HPLC chromatograms of gallic acid, caffeine and catechins; (**B**) HPLC chromatograms of procyanidin B2 (PB2); (**C**) Total ion chromatogram of CLE (**lower**), ion chromatogram of CLE at *m*/*z* 289.071 (**middle**) and ion chromatogram of CLE at *m*/*z* 577.134 (**upper**) at ESI^−^; (**D**) Total ion chromatogram of the PB2 standard (**lower**) and ion chromatogram of the PB2 standard at *m*/*z* 577.134 (**upper**); (**E**) ESI^−^-MS spectra of peaks at retention time 2.718 min for PB2 (**lower**), 2.589 min for C (**middle**), and 2.946 min for EC (**upper**), respectively; (**F**) ESI^−^-MS/MS spectra of peaks at retention time 2.768 min for PB2 (**lower**), 2.611 min for C (**middle**), and 2.996 min for EC (**upper**), respectively; (**G**) ESI^−^-MS/MS spectra of the peak at retention time 2.718 min for the PB2 standard (**lower**) and ESI^−^-MS spectra of the peak at retention time 2.711 min for the PB2 standard (**upper**).

The general chemical analysis (Table 1) revealed that almost half of CLE was composed of polyphenols. Surprisingly, the polyphenol content of CLE was even higher than that of most green tea water extracts. Soluble sugars and saponins were the second and third most abundant compounds, respectively. High performance liquid chromatography (HPLC) analysis, together with UPLC-Q-TOF/MS analysis, confirmed that epicatechin (EC) and procyanidin B2 (PB2, or its isomers) were predominant polyphenols in the CLE (Figure 2) (For detailed information about the identification of EC and PB2, please see Supplementary Materials). EC and PB2 (including isomers) accounted for approximately 60% (*w*/*w*) and 17% (*w*/*w*) of total polyphenols in CLE, respectively (Table 2). Gallic acid and caffeine were not detected in the extract (Figure 2). The above results suggested that CLE was an excellent source of polyphenols, especially EC and PB2 (including isomers).

### 2.2. Antibacterial Activities of CLE

Periodontitis and pharyngitis belong to infectious diseases. Periodontitis is a set of inflammatory diseases that affects the tissues surrounding the teeth, which can lead to loosening and even loss of teeth and fetid breath. Pharyngitis is a type of upper respiratory tract infection, typically resulting in a sore throat and fever. *Porphyromonas gingivalis* and β-hemolytic *Streptococcus* are principal pathogens of periodontitis and pharyngitis, respectively [4,5]. In this study, the inhibitory effects of CLE against *Porphyromonas gingivalis*, β-hemolytic *Streptococcus*, and two common opportunistic pathogenic bacteria, namely *Staphylococcus aureus* and *Escherichia coli*, were tested. According to the results, the minimum inhibitory concentrations (MICs) of CLE against *Porphyromonas gingivalis*, β-hemolytic *Streptococcus*, *Staphylococcus aureus*, and *Escherichia coli* were 0.625, 1.25, 2.5, and 1.25 mg/mL, respectively (Table 3).

Previous studies have revealed that EC and procyanidins exhibit antibacterial properties. EC-enriched *Fagopyrum cymosum* extracts were capable of inhibiting β-hemolytic *Streptococcus* and *Streptococcus pneumoniae* in vitro and in vivo [6]. Proanthocyanidin-enriched grape seed extract inhibited the endotoxin release of *Porphyromonas gingivalis* and the MIC of the extract against *Porphyromonas gingivalis* was 0.8 mg/mL [7]. Here, we proved that the MICs of EC against *Porphyromonas gingivalis*, β-hemolytic *Streptococcus*, *Staphylococcus aureus*, and *Escherichia coli* were 1.25, 1.25, 1.25, and 1.25 mg/mL, respectively (Table 4). No obvious antibacterial effects of PB2 were detected under the experimental conditions, indicating that the MICs of PB2 against these four bacteria was greater than 1.25 mg/mL, respectively (Table 4). This hinted that EC might be responsible for the antibacterial activities of CLE.

The MIC of EC against *Porphyromonas gingivalis* was higher than that of CLE, suggesting the possibility of the presence of other antibacterial components in the CLE. Based on the chemical analysis, CLE contained substantial amounts of saponins. Saponins are strong surfactant agents and can interrupt the stability of the cell membrane and increase cell leakage, leading to the death of bacteria. Saponins derived from various plants have been shown to exhibit antibacterial activities, even at very low concentrations [8]. Thus, saponins present in CLE might also possess antibacterial properties. Nevertheless, further studies are needed to verify this.

### 2.3. Anti-Inflammatory Activities of CLE

**Figure 3:**
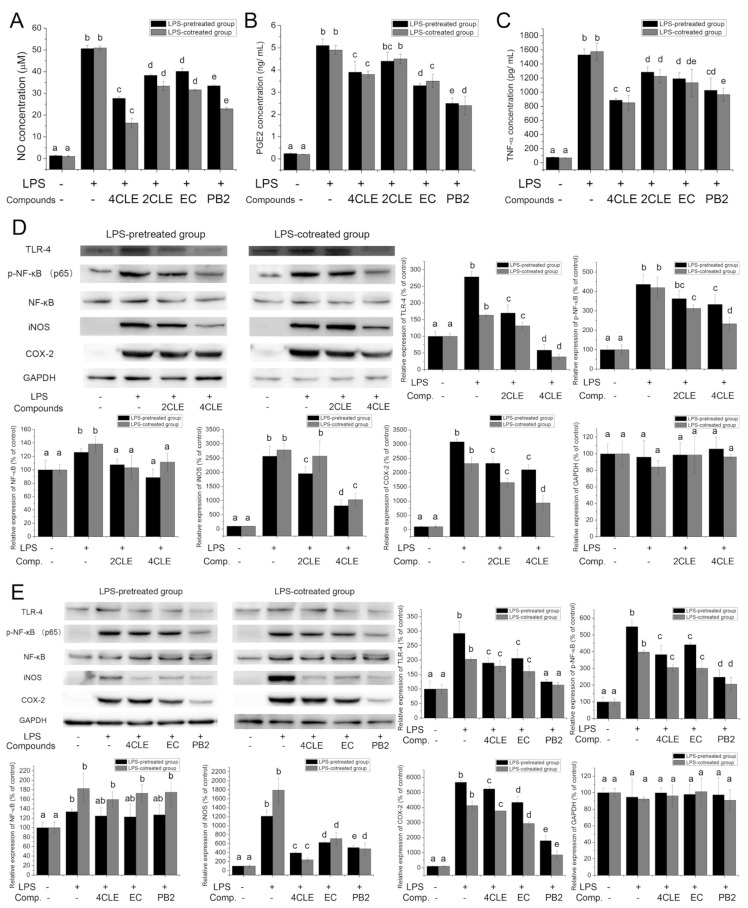
Anti-inflammatory activities of CLE, EC, and PB2. (**A**–**C**) The effects on nitric oxide (NO), prostaglandin E2 (PGE2), and tumor necrosis factor α (TNF-α) secretion of LPS-pretreated or co-treated RAW 264.7 cells; (**D**,**E**) The impacts on the expression of toll-like receptor 4 (TLR4), p-NF-κB (p65), NF-κB (p65), inducible nitric oxide synthase (iNOS), and cyclooxygenase-2 (COX-2). Glyceraldehyde-3-phosphate dehydrogenase (GAPDH) served as the loading control. The same letter within each column indicates no significant difference (*p* > 0.05). Comp. is short for compounds. 4CLE (2CLE) is short for 400 (200) μg/mL CLE. The dosages of EC and PB2 were identical amount of EC and PB2 in 400 μg/mL CLE, which were 120 μg/mL and 34.4 μg/mL, respectively.

Lipopolysaccharide (LPS) is a prototypical endotoxin and induces a strong response from normal animal immune systems. It binds the toll-like receptor 4 (TLR-4), activates inflammatory signaling pathways, promotes the secretion of pro-inflammatory cytokines, nitric oxide (NO) and eicosanoids in several types of immune cells, and finally causes classical symptoms of inflammation. Nuclear factor-κB (NF-κB) is one of the downstream proteins in the TLR-4 pathway. Activation of NF-κB leads to the transcription of inducible nitric oxide synthase (iNOS) and cyclooxygenase-2 (COX-2), and these two proteins are key enzymes involved in the production of NO and prostaglandins, respectively. To evaluate the anti-inflammatory activities of CLE, RAW264.7 cells were treated with LPS and CLE, and then the expression of TLR-4 pathway-related proteins and the release of NO, prostaglandin E2 (PGE2), and tumor necrosis factor α (TNF-α) were determined. The results elucidated that CLE decreased the release of NO, PGE2, and TNF-α (Figure 3A–C). CLE also lowered the levels of TLR-4, p-NF-κB (p65), iNOS, and COX-2 (Figure 3D). This implied that CLE suppressed LPS-stimulated inflammation by inactivating the TLR-4/NF-κB/iNOS and TLR-4/NF-κB/COX-2 pathways.

PB2, a B type proanthocyanidin widely present in plants (e.g., grape seeds, apples, cocoa beans, litchi, and hawthorn), displays anti-inflammatory activities in many ways [9,10,11]. It markedly elevated the expression of a TLR-4 negative regulator, thus blocking LPS-induced expression of cell surface molecules, activation of mitogen-activated protein kinases (MAPKs, e.g., ERK1/2, p38, JNK), translocation of NF-κB (p65), and production of pro-inflammatory cytokines in macrophages [12]. By preventing the activation of NF-κB and MAPKs, PB2 also suppressed the expression of COX-2 [13]. Similar to PB2, EC also exerts anti-inflammatory activities [11]. It inhibited the production of NO, PGE2, TNF-α, and interleukin-6 in LPS-induced macrophages by inactivation of the NF-κB, MAPKs, and JAK2/STAT3 pathways [14]. In this research, PB2 and EC at concentrations identical to the concentrations of PB2 and EC in 400 μg/mL CLE were demonstrated to decrease LPS-stimulated up-regulation of TLR4, p-NF-κB (p65), COX-2, iNOS, NO, PGE2, and TNF-α (Figure 3) in RAW 264.7 cells. Compared with EC, PB2 showed much more potency in suppressing the LPS-stimulated inflammatory response. It indicated that PB2 was vital for the anti-inflammatory activities of CLE while EC played a subordinate role.

### 2.4. Antioxidant Activities of CLE

Inflammation stimulates cellular reactive oxygen species (ROS) production [15]. Excessive ROS in turn enhance inflammation. In addition, overproduction of ROS causes injury and even necrosis of macrophages, resulting in an uncontrolled release of cell contents into the extracellular space, which deteriorates inflammation.

**Figure 4:**
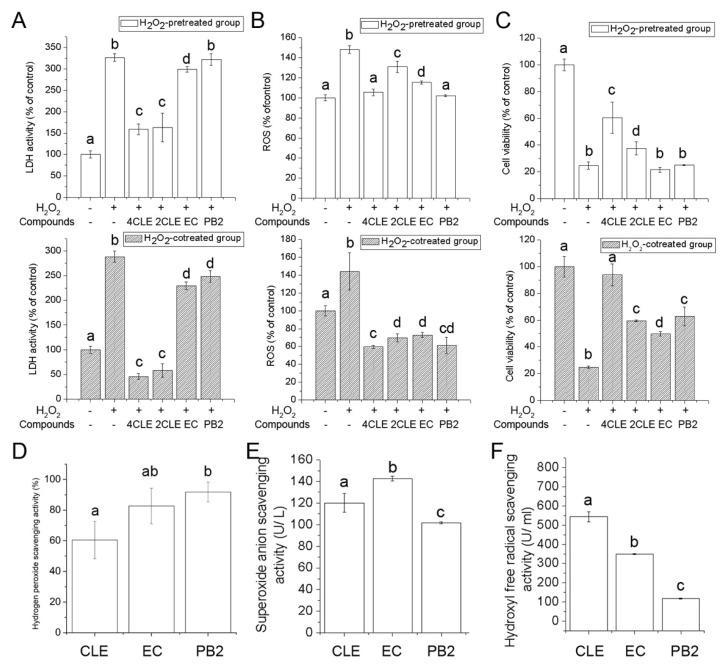
Antioxidant activities of CLE, EC, and PB2. (**A**) The effects on the lactate dehydrogenase (LDH) release of 0.5 mM H_2_O_2_-pretreated or co-treated RAW 264.7 cells; (**B**) The effects on the intracellular reactive oxygen species (ROS) levels of 0.5 mM H_2_O_2_-pretreated or co-treated RAW 264.7 cells; (**C**) The effects on the viability of 1 mM H_2_O_2_-pretreated or co-treated RAW 264.7 cells; (**D**–**F**) The hydrogen peroxide and free radical scavenging activities of CLE, EC, and PB2. The same letter within each column indicates no significant difference (*p* > 0.05). 4CLE (2CLE) is short for 400 (200) μg/mL CLE. The dosages of EC and PB2 were identical amount of EC and PB2 in 400 μg/mL CLE, which were 120 μg/mL and 34.4 μg/mL, respectively.

In this study, the effects of CLE on rescuing or protecting cells from oxidative stress-induced damage were investigated. Lactate dehydrogenase (LDH) is an enzyme found in almost all living cells and is released when cell damage occurs [16]. By measuring the LDH activities in the cell culture supernatant, the severity of H_2_O_2_-induced cell damage can be determined. According to the results, CLE decreased 0.5 mM H_2_O_2_ caused-cell damage in pretreated and cotreated models (Figure 4A). CLE was more potent in preventing H_2_O_2_-mediated cell damage than in rescuing it. Intracellular ROS levels were also measured. A cell-permeable non-fluorescent probe, 2′,7′-dichlorodihydrofluorescein diacetate (DCFH-DA), is hydrolyzed by mitochondrial esterase to form DCFH, which is then oxidized by ROS to form the cell-impermeable fluorescent compound 2′,7′-dichlorofluorescein (DCF). In this study (Figure 4B), CLE significantly inhibited H_2_O_2_-induced increase of intracellular ROS levels.

When damage caused by oxidative stress is too severe to be repaired, cells can no longer remain living. The MTT assay is a colorimetric assay used to measure the number of living cells. Results from the MTT assay illustrated that the viability of 1 mM H_2_O_2_-pretreated and co-treated cells was significantly increased by CLE, suggesting that CLE could reduce the cytotoxicity of H_2_O_2_ (Figure 4C). Compared with the H_2_O_2_-pretreated group, the viability of the H_2_O_2_-cotreated group was significantly higher. The results of the in vitro free radical-scavenging tests (Figure 4D–F) suggested that one of the possible reasons was that CLE could directly react with H_2_O_2_ to lower the concentration of H_2_O_2_ in the culture medium and CLE was capable of scavenging free radicals, such as the superoxide anion and hydroxyl free radical.

Considerable researches have shown that EC and PB2 are effective antioxidants. They not only scavenge free radicals, but also activate antioxidant enzymes [17]. In this research, EC and PB2 were confirmed as contributors to the antioxidant activities of CLE (Figure 4). Although the concentration of PB2 used in these experiments (34.4 μg/mL) was much lower than that of EC (120 μg/mL), their antioxidant activities in cell models were not significantly different. Considering that the free radical scavenging activity of PB2 was weaker than EC at the concentration used in this experiment, it could be assumed that PB2 exhibited its antioxidant activity via mechanisms other than directly reacting with free radicals, possibly by having an impact on antioxidant enzymes. According to previous studies, PB2 was capable of increasing the activities of antioxidant enzymes, including superoxide dismutase (SOD), catalase (CAT), and glutathione peroxidase (GSH-Px) [18,19]. Furthermore, it was noteworthy that the antioxidant activities of CLE were much higher than those of EC or PB2 in cell models, suggesting that there were other antioxidant components existing in CLE. Previous research has illustrated that some plant-derived polysaccharides and saponins have antioxidant activities and are able to protect cells from oxidative damage [20,21]. The saponins and polysaccharides present in CLE might also have antioxidant activities. However, the roles of these compounds have not been studied in the present research and further work is needed to determine their functions.

### 2.5. Acute Oral Toxicity of CLE

Acute oral toxicity test showed that no mice died during the observation period after a single administration of CLE at the dosage of 20 g/kg body weight (Table 5). No acute poisoning symptoms were observed during the observation period. No abnormalities of organs in size, color, shape, consistency or other characteristics were detected by the gross examination. According to the Chinese Standard of GB 15193.3-2014, CLE was regarded as practically non-toxic.

### 2.6. Effects of CLE, EC and PB2 on Normal RAW 264.7 Cells

The effects of CLE, EC, and PB2 on RAW 264.7 cells without the presence of LPS and H_2_O_2_ were also studied. Generally, CLE, EC, or PB2 alone at the test concentration didn’t significantly affect the viability, LDH activities, ROS levels, and inflammatory-related pathways of RAW 264.7 cells. These results were presented in the Supplementary Materials.

## 3. Materials and Methods

### 3.1. Materials

The buds of *Castanopsis lamontii* were collected in the Dehong Area, Mang City, Yunnan Province, China (24°17′12″ N, 98°23′6″ E) and identified by Botanist Keke Chen, Kunming Institute of Botany, Chinese Academy of Sciences. The hydroxyl free radical assay kit and superoxide anion assay kit were obtained from Nanjing Jiancheng Bioengineering Institute. The LDH cytotoxicity assay kit, NO assay kit, primary antibodies (p-NF-κB (p65) (Product #AN371), NF-κB (Product #AF0246), GAPDH (Product #AF0006), and secondary antibodies (Product #A0216 & A0208) were purchased from Beyotime Institute of Biotechnology, Co. Ltd. (Haimen, China) Primary antibodies (TLR4 (Product #14358S), COX-2 (Product #12282), iNOS (13120S) were purchased from Cell Signaling Technology, Co. Ltd. (Danvers, MA, USA). The MTT cell proliferation and cytotoxicity assay kit was purchased from Nanjing KeyGEN Bio-TECH, Co. Ltd. (Nanjing, China) The PGE2 ELISA kit and the TNF-α ELISA kit were purchased from Jiangsu Meimian industrial Co., Ltd. (Zhangjiagang, China) Chemical standards used in HPLC analysis, UPLC-Q-TOF/MS analysis and cell assays were purchased from Sigma-Aldrich Co., Ltd. (St. Louis, MO, USA).

### 3.2. Preparation of CLE

The buds of *Castanopsis lamontii* were dried, powdered, and filtered through a 60-mesh screen. The powder was extracted with distilled water (powder weight: water volume = 1:100) for 20 min at 80 °C and centrifuged at 8000 rpm for 10 min to remove powder residues. The supernatant was collected and vacuum dried.

### 3.3. Determination of Chemical Composition

The total phenolic, free amino acid, and water extract contents were measured according to the Chinese Standard of GB/T 8313-2008, GB/T 8314-2013 and GB/T 8305-2013, respectively.

The total flavonoid content was determined using the Dowd method with modifications. Briefly, 10 mL 1% aluminium trichloride (AlCl_3_) was mixed with 0.5 mL sample solution and incubated at room temperature for 10 min. The absorbance was measured at 420 nm using a UV-VIS-NIR spectrophotometer (UV-3600, Shimadzu Co., Ltd., Kyoto, Japan).

The total soluble sugar content was estimated using the anthrone-sulfuric acid colorimetric method. Briefly, 1 mL sample solution was mixed with 4 mL anthrone-sulfuric acid (2 mg/mL) and reacted at 100 °C for 10 min. After cooling to room temperature, the absorbance was read at 620 nm.

To measure the polysaccharide content, the sample solution was mixed with 95% ethanol (*v*/*v* = 1:5), maintained at 4 °C overnight, and centrifuged to spin down the polysaccharides. Samples were then analyzed using the anthrone–sulfuric acid method.

The protein content was measured using the Bradford method [22].

To determine the total saponin content [23], the sample solution was extracted three times with water-saturated n-butanol. The n-butanol layer was collected and washed three times with ammonia water. Then, the n-butanol layer was dried and the residues were dissolved in methanol for measurement. A 200 μL sample solution was added to 0.5 mL 8% vanillin-alcohol solution and 3 mL 70% sulfuric acid and the reaction system was incubated at 60 °C for 10 min. After cooling to room temperature, the absorbance was read at 540 nm.

### 3.4. HPLC Analysis

The catechins, gallic acid, and caffeine contents were analyzed according to a previously published method [24].

The PB2 content was examined using a previously reported method which could distinguish eight B-type procyanidin dimers [25].

### 3.5. UPLC-Q-TOF/MS Analysis

An UPLC-Xevo G2-S Q-TOF/MS system (Waters, Milford, MA, USA), equipped with an AC-QUITY UPLCTM I-class solvent manager, an ACQUITY UPLCTM sample manager-FTN, an AC-QUITY UPLCTM column manager, and a Xevo G2-S Q-TOF/MS with a Zspray electrospray ion source at real time mass correction, was used for the determination. Data collection and system control was performed using the Waters MassLynx 4.1 software workstation (Waters Corporation, Milford, MA, USA).

The UPLC HSS T3 column (100 mm × 2.1 mm, 1.7 µm, Waters, Milford, MA, USA) was used for the separation. The gradient separation was carried out using 0.1% formic acid in water and 0.1% formic acid in acetonitrile as mobile phases A and B, with the flow rate at 0.4 mL/min for 30 min and the column temperature at 40 °C. When the gradient elution changed from 0 to 2 min, B increased lin-early from 5% to 20%; from 2 to 3.3 min, B was 20%; from 3.3 to 5 min, B increased linearly from 20% to 30%; from 5 to 7 min, B increased linearly from 30% to 40%; from 7 to 10 min, B increased linearly from 40% to 50%; from 10 to 12 min, B increased linearly from 50% to 100%; from 12 to 14.5 min, B was 100%; from 14.5 to 14.6 min, B reduced linearly from 100% to 5%; from 14.6 to 16 min, B was 5%.

The Q-TOF/MS analyses were performed under the positive and negative ionization full scan modes with a capillary electrospray voltage of 3 kV and 1.5 kV, respectively, a cone voltage of 40 V, an offset voltage of 80 V, a source temperature of 150 °C, a desolvation gas of nitrogen at 99.95% with the desolvation temperature at 350 °C and a flow rate of 800 L/h, and a cone gas of nitrogen at 99.95% with a flow rate of 50 L/h. The scan range and acquisition rate was at *m*/*z* 50–1200 and 2 spectra/s. The calibration reference ion was at *m*/*z* 556.2771 for ESI^+^ and *m*/*z* 554.2665 for ESI^−^.

### 3.6. Determination of Minimum Inhibitory Concentrations (MIC) against Bacteria

The MICs against *Porphyromonas gingivalis* (ATCC 33277), β-hemolytic *Streptococcus* (CMCC (B) 32210), *Staphylococcus aureus* (CMCC (B) 26003) and *Escherichia coli* (CMCC (B) 44102) were determined using the micro-dilution method [26].

### 3.7. Determination of Anti-Inflammatory Activities in LPS-Treated RAW264.7 Cells

Mouse macrophage RAW264.7 cells were seeded into 96-well plates at a density of 1.5 × 10^4^ per well in RPMI-1640 medium incorporating 10% fetal bovine serum (FBS). Cells were grown in a humidified incubator containing 5% CO_2_ at 37 °C. After overnight growth, cells were treated with 500 ng/mL LPS for 2 h prior to adding 200 μg/mL CLE, 400 μg/mL CLE, 120 μg/mL epicatechin (EC) (equivalent to the concentration of EC in 400 μg/mL CLE), or 34.4 μg/mL PB2 (equivalent to the concentration of PB2 in 400 μg/mL CLE) for 24 h to test the protective activity. Cells were co-treated with 500 ng/mL LPS and CLE/EC/PB2 for 24 h to test the rescuable activity. The NO, PGE2, and TNF-α concentrations were measured using the NO assay kit, PGE2 ELISA kit, and TNF-α kit, respectively.

### 3.8. Western Blot

The Western blot assay was conducted using a previously published method [27]. GAPDH served as the loading control.

### 3.9. Determination of Antioxidant Activities in Hydrogen Peroxide-Treated RAW264.7 Cells

RAW264.7 cells were seeded into 96-well plates at a density of 1.5 × 10^4^ per well in RPMI-1640 medium incorporating 10% FBS. After overnight growth, cells were treated with 0.5 mM hydrogen peroxide (H_2_O_2_) for 1 h prior to adding 200 μg/mL CLE, 400 μg/mL CLE, 120 μg/mL EC (equivalent to the concentration of EC in 400 μg/mL CLE), or 34.4 μg/mL PB2 (equivalent to the concentration of PB2 in 400 μg/mL CLE) for 24 h to test the protective activities. Cells were co-treated with 0.5 mM H_2_O_2_ and CLE/EC/PB2 for 24 h to test the rescuable activity. Then the LDH release assay were conducted using the LDH cytotoxicity assay kit, according to the manufacturer’s instructions. The production of intracellular ROS was monitored by a multi-functional microplate reader (Thermo Scientific Varioskan Flash, Waltham, MA, USA) after the cells were incubated with 2′,7′-dichlorofluorescin diacetate (DCHF-DA). To evaluate the protective or rescuable activity of CLE against oxidative stress-induced cell death, RAW 264.7 cells were treated with 1 mM H_2_O_2_ for 1 h prior to adding CLE/EC/PB2, or co-treated with 1 mM H_2_O_2_ and CLE/EC/PB2 for 24 h. Then the cell viability was assessed using a MTT cell proliferation assay kit.

### 3.10. Determination of Free Radical Scavenging Activities

Hydrogen peroxide scavenging activity, superoxide anion-scavenging activity, and hydroxyl free radical-scavenging activity were measured using a hydrogen peroxide assay kit, a superoxide anion assay kit, and a hydroxyl free radical assay kit according to the manufacturer’s instructions, respectively.

### 3.11. Acute Oral Toxicity Tests

Ten female and ten male ICR mice (body weight 18–22 g) were used for the acute oral toxicity tests (License No. SCXK (Zhe) 2014-0008 and SYXK (Zhe) 2014-0008). The tests were carried out according to the Chinese Standard GB 15193.3-2014. Briefly, the mice were housed in a controlled environment (12 h light-dark cycle, temperature of 22 ± 2 °C, relative humidity of 50% ± 10%, standard rodent diet). Prior to the administration, mice were fasted for 6 h with free access to water. Each mouse was administrated with CLE at the dosage of 20 g/kg body weight via intragastic gavage. Three hours later, mice were allowed free access to food and water. The body weights, clinical appearances (e.g., behavior modification, skin lesions, and feces) and survival rates of tested mice were recorded in the next 14 days. At the end of the observation, mice were sacrificed after a 12 h fasting period and dissected for gross examination (including the organs’ size, color, consistency, and other characteristics). Based on the GB 15193.3-2014, test material with median lethal dose (LD50) ≥ 20 g/kg is defined as practically non-toxic.

### 3.12. Statistical Analysis

The data are presented as means ± standard error of the means (SEM). All experiments were carried out in triplicate and repeated in three independent sets of experiments. The results were analyzed with SPSS Version 18.0 for Windows using one-way analysis of variance (ANOVA) and post hoc tests (2-sided Dunnett’s test) to determine both overall differences and specific differences between each treatment and control. *p*-values < 0.05 were considered statistically significant.

## 4. Conclusions

This research revealed that polyphenols, soluble sugars, and saponins accounted for approximately 85% (*w*/*w*) of CLE. CLE had excellent antibacterial, anti-inflammatory, and antioxidant activities. EC and PB2 were the key bioactive components in this extract. EC, in particular, contributed to the antibacterial activities of CLE, while PB2 mainly contributed to the anti-inflammatory activities. Both compounds were responsible for the antioxidant activities. Acute oral toxicity tests confirmed that CLE was practically non-toxic. These results provide experimental evidence of the health-beneficial effects of CLE, and indicate the potential for application of CLE as a functional food additive and health supplement in the food and health industries.

## Figures and Tables

**Table 1:** Chemical compositions in the water extract from the buds of *Castanopsis lamontii* (CLE).

Components	Contents (%)
Polyphenols	48.38 ± 0.192
Soluble sugars	24.43 ± 1.944
Saponins	12.19 ± 0.312
Amino acids	2.520 ± 0.168
Polysaccharides	2.184 ± 0.048
Proteins	1.440 ± 0.240
Flavonoids	1.056 ± 0.000
Caffeine	Not detected

**Table 2:** Concentrations of catechins, procyanidin B2, and gallic acid in CLE solution (400 μg/mL).

Components	Concentrations (μg/mL)
Epicatechin (EC)	120.1 ± 1.1
Procyanidin B2 (PB2) (including isomers)	34.4 ± 3.2
Catechin (C)	9.6 ± 0.5
Gallic acid (GA)	Not detected
Gallocatechin (GC)	0.1 ± 0.0
Epigallocatechin (EGC)	0.5 ± 0.0
Epigallocatechin gallate (EGCG)	0.3 ± 0.0
Gallocatechin gallate (GCG)	0.1 ± 0.0
Epicatechin gallate (ECG)	0.1 ± 0.0
Catechin gallate (CG)	0.2 ± 0.0

**Table 3:** Minimum inhibitory concentrations (MICs) of CLE against pathogens and opportunistic pathogens.

Concentrations of CLE (mg/mL)	0	0.156	0.313	0.625	1.25	2.50	5.00	10.0
*Porphyromonas gingivalis*	+	+	+	-	-	-	-	-
β-hemolytic *Streptococcus*	+	+	+	+	-	-	-	-
*Staphylococcus aureus*	+	+	+	+	+	-	-	-
*Escherichia coli*	+	+	+	+	-	-	-	-

“+” means visible growth of the bacteria, while “-” means no visible growth of the bacteria.

**Table 4:** The MICs of EC and PB2 against pathogens and opportunistic pathogens.

**Concentrations of EC (mg/mL)**	**0**	**0.078**	**0.156**	**0.313**	**0.625**	**1.25**	**2.50**	**5.00**
*Porphyromonas gingivalis*	+	+	+	+	+	-	-	-
β-hemolytic *Streptococcus*	+	+	+	+	+	-	-	-
*Staphylococcus aureus*	+	+	+	+	+	-	-	-
*Escherichia coli*	+	+	+	+	+	-	-	-
**Concentrations of PB2 (mg/mL)**	**0**	**0.020**	**0.039**	**0.078**	**0.156**	**0.313**	**0.625**	**1.25**
*Porphyromonas gingivalis*	+	+	+	+	+	+	+	+
β-hemolytic *Streptococcus*	+	+	+	+	+	+	+	+
*Staphylococcus aureus*	+	+	+	+	+	+	+	+
*Escherichia coli*	+	+	+	+	+	+	+	+

“+” means visible growth of the bacteria, while “-” means no visible growth of the bacteria.

**Table 5:** Acute oral toxicity of CLE.

Gender	Initial Average Body Weight (g)	Final Average Body Weight (g)	Death	LD_50_ (g/kg)
Female	20.6 ± 1.3	28.7 ± 1.5	0	>20.0
Male	20.1 ± 1.2	30.4 ± 1.6	0	>20.0

## Supplementary Materials

The supplementary materials provide information about the detailed process of identification of EC and PB2, as well as the effects of CLE, EC and PB2 on normal RAW 264.7 cells. The following are available online, Figure S1: Effects of CLE, EC and PB2 on normal RAW 264.7 cells.
