# Supplementary material for: Castanopsis lamontii Water Extract Shows Potential in Suppressing Pathogens, Lipopolysaccharide-Induced Inflammation and Oxidative Stress-Induced Cell Injury
Source: Molecules. 2019 Jan 12;24(2):273. doi: 10.3390/molecules24020273 (PMC6359260; doi:10.3390/molecules24020273)
Supplement: Supplementary file 1 [file molecules-24-00273-s001.pdf]

Supplementary Materials:

1. Identification of C and EC

First, HPLC-UV analysis revealed that there were obvious peaks presenting at 11.4 and 16.5 min in the HPLC chromatogram of CLE (Figure 2A lower), whose retention time was identical to the retention time of the C and EC standard, respectively (Figure 2A upper). It indicated that CLE probably contained C and EC.

UPLC-Q-TOF/MS was used for further identification. As shown in Figure 2C lower, there were obvious peaks presenting at 2.59, 2.70, and 2.93 min in the total ion chromatogram of CLE. Based on the total ion chromatogram of CLE at *m*/*z* 289.071 (Figure 2C middle), there were peaks at 2.59 and 2.93 min. ESI^--MS spectra of peaks at 2.589 and 2.946 min revealed that the predominant peak occurred at *m*/*z* 289.071, respectively (Figure 2E middle and upper). It matched the predicted *m*/*z* of C and EC at ESI^- mode. ESI^--MS/MS spectra of peaks at 2.611 and 2.996 min further confirmed it (Figure 2F middle and upper).

2. Identification of PB

UPLC-Q-TOF/MS was used for identification. There was a peak presenting at 2.69 min in the total ion chromatogram of the PB2 standard (Figure 2D lower). So was the ion chromatogram of the PB2 standard at *m*/*z* 577.135 (Figure 2D upper). Correspondingly, there was a peak presenting at 2.70 min in the total ion chromatogram of CLE (Figure 2C lower). Based on the ion chromatogram of CLE at *m*/*z* 577.134 (Figure 2C upper), the main peak occurred at 2.70 min. ESI^--MS spectra of the peak at 2.718 min revealed that the predominant peak occurred at *m*/*z* 577.134 (Figure 2E lower). It matched the predicted *m*/*z* of PB2 at ESI^- mode. And it matched the ESI^--MS spectra of the PB2 standard (Figure 2G upper). In addition, the ESI^--MS/MS spectra of the peak at 2.768 min (Figure 2F lower) was consistent with the ESI^--MS/MS spectra of the PB2 standard (Figure 2G lower).

Since there are eight isomeric B-type procyanidin dimers and mass spectroscopy perhaps can't differentiate them, a previously reported HPLC-UV method which could distinguish them was applied. The HPLC-UV analysis revealed that there was an obvious peak presenting at 30.007 min in the HPLC chromatogram of CLE (Figure 2B lower), whose retention time was identical to the retention time of the PB2 standard (Figure 2B upper). It indicated that CLE contained PB2.

Quantification based on the result of HPLC indicated the concentration of PB2 in 400 μg/mL CLE was 31.6 μg/mL. Quantification based on the result of UPLC-Q-TOF/MS indicated the concentration of PB2 in 400 μg/mL CLE was 34.4 μg/mL. Therefore, PB2 is considered to be the predominant procyanidin dimer in CLE.

3. Effects of CLE, EC and PB2 on Normal RAW 264.7 Cells

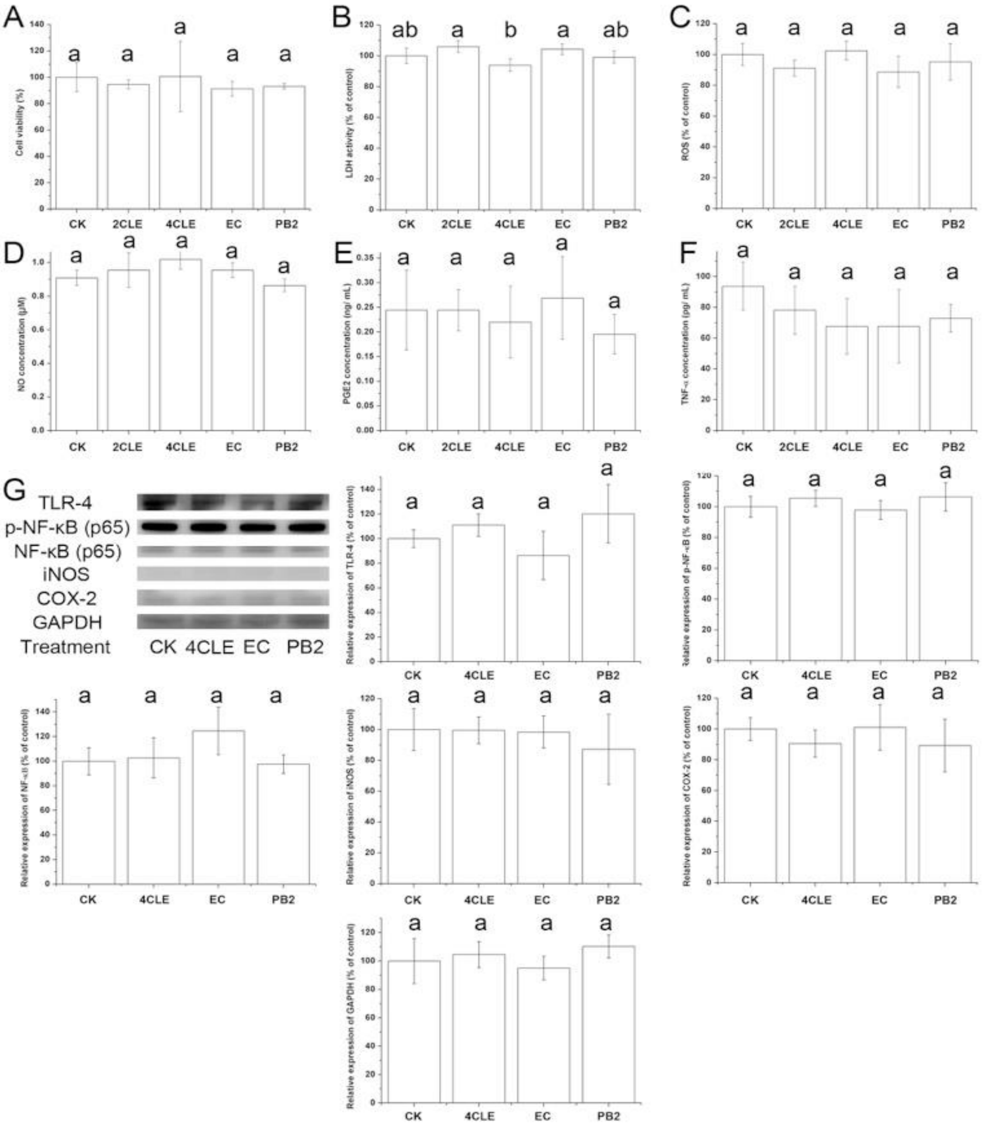

Figure S1. Of CLE, EC, and PB2 on normal RAW 264.7 cells. The cell viability (**A**), LDH activities (**B**), ROS levels (**C**), NO levels (**D**), PGE2 levels (**E**), TNF-α levels (**F**), and protein levels of pro-inflammatory pathways (**G**) of RAW 264.7 cells only exposed to investigated substances. The same letter within each column indicates no significant difference ($p > 0.05$). 4CLE (2CLE) is short for 400 (200) μg/mL CLE. The dosages of EC and PB2 were identical amount of EC and PB2 in 400 μg/mL CLE, which were 120 μg/mL and 34.4 μg/mL, respectively.
